# The Perception of Facial Emotional Change in Social Anxiety: An ERP Study

**DOI:** 10.3389/fpsyg.2018.01737

**Published:** 2018-09-28

**Authors:** Qi Zhang, Guangming Ran, Xueping Li

**Affiliations:** ^1^College of Preschool and Primary Education, China West Normal University, Nanchong, China; ^2^Department of Psychology, Institute of Education, China West Normal University, Nanchong, China

**Keywords:** angry-neutral facial emotional changes, neutral-angry facial emotional changes, social anxiety, perception, ERP

## Abstract

Social anxiety is one of the psychological symptoms that most commonly occur in social interaction. Although previous behavioral studies have investigated the neutral-angry facial emotion change in social anxiety, none of the previous studies have, however, directly investigated the angry-neutral facial emotional change. Furthermore, less is known about the neural correlates of the facial emotion changes in individuals with social anxiety. The main goal of the present study was to explore the perception of facial emotional changes in individuals with social anxiety, using high temporal resolution event-related potential techniques. Behaviorally, accuracy in the angry-neutral facial emotional change trail was lower than that in the neutral-neutral case. Neurally, we found that the N170 amplitudes in angry-neutral facial emotional change trial were larger than those in the neutral-neutral case for high social anxiety (HAS) participants, probably reflecting that they might engage in more analytical processing of different facial elements. Interestingly, HSA participants showed smaller P200 left hemisphere amplitudes in the angry-neutral facial emotional change trial when compared with the neutral-neutral case, which suggested that they might have difficulties in processing emotions when they encounter these facial emotional changes. Finally, the late positive potential amplitudes in the neutral-angry and angry-neutral facial emotional change trials were smaller than those in the neutral-neutral case, regardless of the social anxiety. These results suggest that social anxiety influences the facial emotional changes mainly at an earlier stage of processing.

## Introduction

Social anxiety refers to a state of anxiety that results from the presence of interpersonal evaluation in social interaction (Pierce, 2009). Although social anxiety is a milder form of social phobia, it is one of the most common forms of anxiety (Pierce, 2009; Kross et al., 2014). It is associated with poor interpersonal relations, such as negative, aversive, or exclusionary experiences with others (Mathew et al., 2011). It begins during one’s childhood or adolescence, with a mean age of onset between 14 and 16 years (Iverach and Rapee, 2014). Individuals with high social anxiety (HSA) show enhanced vigilance to stimuli associated with social threat, such as angry faces (Kirsch, 2015).

As fundamental emotional stimuli, emotional faces play a significant role in our daily life (Ran et al., 2014b). A large number of electrophysiological studies have explored the perception of emotional faces in individuals with social anxiety. An early event-related potential (ERP) component is P100, which is probably generated by the extrastriate cortex (Campanella et al., 2006). Such a component is a positive wave that is recorded around 100 ms following the stimulus presentation onset at occipital sites (Fishman et al., 2012). The P100 component is thought to reflect early visual processing (Schendan et al., 1998; Taylor and Khan, 2000). A set of ERP studies on facial processing in individuals with HSA showed increased P100 amplitudes in response to threatening/angry faces (Mühlberger et al., 2009; Peschard et al., 2013). Interestingly, Rossignol et al. (2012) reported enhancement of the P100 amplitudes in individuals with HSA for all emotional faces rather than a specific increase in response to threatening faces.

Unlike the P100 component, the N170 component (a negative wave), is recorded at occipitotemporal sites peaking roughly at 170 ms after stimulus onset (Bentin et al., 1996; Chen et al., 2015). A meta-analysis of N170 shows that this negative component is usually observed in experiments that use faces as stimuli (Hinojosa et al., 2015). It has previously been argued that N170 reflects the analytical processing of different facial elements (Peschard et al., 2013). There is evidence that N170 is larger (more negative amplitudes) for HSA individuals when they perform a task that measures the processing of emotional faces that are threatening (Wieser et al., 2010; Ran and Chen, 2017). However, several other studies have failed to find the moderating effect of social anxiety on N170 (Mühlberger et al., 2009; Li et al., 2017). These discrepancies might stem from differences in participants’ task and experimental design. In the study conducted by Mühlberger et al. (2009), the processing of emotion was implicit. However, participants were explicitly instructed to process emotion in the study conducted by Ran and Chen (2017). More precisely, HSA and low social anxiety (LSA) participants attend to emotion in the explicit task, while they do not attend to emotion in the implicit task, which may affect their configural encoding of emotional faces.

The P200 component, which follows N170 and is recorded at occipitotemporal sites, is the second positive peak in ERP, occurring at around 190 ms to 250 ms after stimulus onset (Sheehan et al., 2005). It is believed to reflect sustained perceptual processing and to be functionally associated with the complexity of emotional appraisal (Peschard et al., 2013). Previous studies that examined the effect of social anxiety on P200 demonstrated increased P200 amplitudes in response to angry faces in HSA individuals (Rossignol et al., 2012). Recently, Ran and Chen (2017) reported that HSA participants exhibited more positive P200 amplitudes in response to angry faces when compared with happy faces.

A late ERP component, which is sensitive to emotional faces, is the late positive potential (LPP) that starts around 300 ms after stimulus onset (Sje and van Strien, 2018). The LPP is a centro-parietal distributed and a positive-going ERP component (Sje and van Strien, 2018). There is evidence that the LPP is larger for emotional stimuli when compared with neutral stimuli (Hagemann et al., 2016). A particular study observed enhanced LPP amplitudes in HSA participants when they viewed angry and disgusted faces (Moser et al., 2008). In addition, a study that explored the affective context found that faces in negative contexts elicit larger LPP amplitudes when compared with faces in neutral contexts in HSA participants (Wieser and Moscovitch, 2015).

Human beings always encounter dynamic but not static emotional faces in daily life (Zhang et al., 2015). Changes in emotional faces are more important to social communication than static emotional faces (Zhang et al., 2015). An earlier study used a morphed faces task to assess participants’ identification biases (Joormann and Gotlib, 2006). The following study adopted the modified morphed faces task to examine dynamic facial expressions in individuals with social anxiety (Heuer et al., 2010). Recently, Gutiérrez-García and Calvo (2017) found that HSA participants were biased towards the interpretation of ambiguous expressions (dynamic facial expressions) in response to threat. Although previous behavioral research has investigated the facial emotional change (neutral-angry facial emotional change: a neutral face suddenly changes to an angry face) in individuals with social anxiety, none of the previous studies have directly investigated the angry-neutral facial emotional change (an angry face suddenly changes to a neutral face). Furthermore, less is known about the neural correlates of the facial emotional changes in individuals with social anxiety.

The main goal of the present study is to explore the perception of facial emotional changes in individuals with social anxiety, using high temporal resolution ERP techniques. As HSA participants show more negative N170 amplitudes when they process threat stimuli (Ran and Chen, 2017), we predict increased N170 amplitudes in the angry-neutral facial emotional change trials. Considering the fact that HSA participants might show an inferior recognition performance with emotional appraisal when they encounter the angry-neutral facial emotional change (Zhang et al., 2015), we hypothesized that they would exhibit decreased P200 amplitudes in the angry-neutral facial emotional change trials. Finally, we expected the LPP amplitudes in the neutral-angry and angry-neutral facial emotional change trials to be smaller than those in the neutral-neutral case.

To test these hypotheses, we adopted a S1-S2 paradigm, which was used by previous researchers to explore the recognition of emotional faces (Ran et al., 2014a, c; Li et al., 2017). Participants with HSA and LSA were instructed to perform a task of facial identity recognition, and their brain responses were recorded using high temporal resolution ERP techniques. It was noted that the participants in present study were students elevated on a scale. They were not clinically diagnosed with social anxiety disorder by a clinician such as a psychiatrist. In this paper, we focus on P100, N170, P200, and LPP components, as it has been proposed that these components are relevant for emotional faces and social anxiety (Peschard et al., 2013; Hagemann et al., 2016; Ran and Chen, 2017).

## Materials and Methods

### Participants

Twenty-seven healthy volunteers (15 women and 12 men; mean age = 21.30 years, standard deviation = 1.84 years) participated in the experiment. They were right-handed and had normal or corrected-to-normal vision. All participants were preselected from a group of 925 undergraduate students based on their social anxiety scores on the Chinese version of the Liebowitz Social Anxiety Scale (LSASC) (He and Zhang, 2004). According to a recent ERP study on individuals with social anxiety (Ran and Chen, 2017), the HSA participants (*N* = 13, 7 women) were defined as those who scored 60 or greater on the LSASC, while the LSA participants (*N* = 14, 8 women) were those who scored under 40. To control comorbidity, all participants were asked to complete the Spielberger State-Trait Anxiety Inventory (Spielberger et al., 1983) and the Beck Depression Inventory (Beck and Beamesderfer, 1974).

It was found that the HSA and LSA participants differed in the LSAS-SR total scores (HSA: 77.23 ± 15.77, LSA: 26.07 ± 6.97; *t*(25) = −11.05, *p* < 0.001), but no group differences were found for age (HSA: 21.16 ± 1.62, LSA: 21.43 ± 2.07; *t*(25) = 0.38, *p* = 0.706), state anxiety level (HSA: 42.08 ± 6.46, LSA: 38.14 ± 7.79; *t*(25) = −1.42, *p* = 0.168), trait anxiety level (HSA: 44.23 ± 7.81, LSA: 40.36 ± 9.39; *t*(25) = −0.86, *p* = 0.397), and depression (HSA: 11.77 ± 6.11, LSA: 8.00 ± 5.88; *t*(25) = −1.63, *p* = 0.115). All participants provided written informed consent and received course credit for their participation. The study was approved by the local ethics committee and the experiments were carried out in accordance with the approved guidelines.

### Materials

**FIGURE 1:**
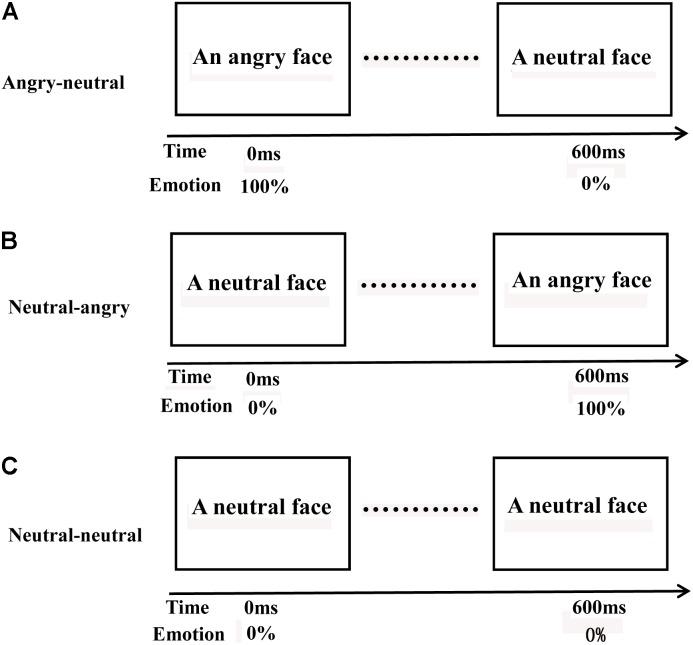
Three different videos of facial emotional changes. Angry-neutral videos **(A)**: an angry face changed to a neutral face; neutral-angry videos **(B)**: a neutral face changed to an angry face; and neutral-neutral videos **(C)**: a neutral face changed to a neutral face.

Following previous studies (Zhang et al., 2017), the present study adopted three different videos of facial emotional changes (*angry-neutral videos* (**Figure 1A**): an angry face changes to a neutral face, *neutral-angry videos* (**Figure 1B**): a neutral face changes to an angry face, *neutral-neutral videos* (**Figure 1C**): a neutral face changes to a neutral face). We used a set of 78 videos (26 angry-neutral videos, 26 neutral-angry videos, and 26 neutral-neutral videos). The videos were created by using the photo-morphing software (Abrosoft FantaMorph). To acquire the neutral-angry videos, pairs of static images displaying a neutral expression (the first image) and an angry expression (the last image) provided reference points for creating a sequential morph between the two images. Sequential morphs were compiled into 600 ms video files (AVI format) with a frame rate of 15 frames per second. The pairs of static images (the first image: an angry expression, the last image: a neutral expression) were employed to create the angry-neutral videos, and the pairs of static images (the first and last image: a neutral expression) were used to acquire the neutral-neutral videos. The faces in the videos had the same ethnicity (Chinese). One half of the videos displayed female faces, while the other half displayed male faces. A group of volunteers, who did not participate in our experiment, were recruited to rate the valence and arousal of the 78 videos on a 9-item scale (1 = low pleasure, low arousal; 9 = high pleasure, high arousal). The videos differed significantly in the valence dimension (*p* < 0.001) but were similar in arousal (*p* = 0.106). The materials also consisted of eight house pictures that were mask stimuli.

### Procedure

**FIGURE 2:**
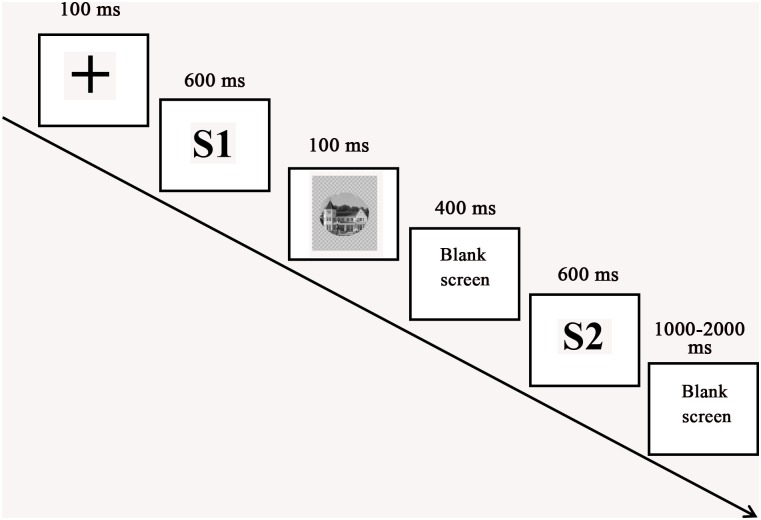
Schematic illustration of the experimental procedure.

Participants were seated comfortably at a position that was 90 cm in front of the computer screen and were instructed to try their best to avoid head movements and eye blinks. The experimental procedure was organized by E-PRIME 2.0. The current experiment employed the S1-S2 paradigm, which was used by previous researchers to explore the recognition of emotional faces (Ran et al., 2014a, c). On each trial of the experiment (**Figure 2**), a fixation cross was shown for 100 ms. After the cross disappeared, the first facial emotional change video (S1) was presented for 600 ms. Next, a stimulus of house picture (a mask stimulus) was depicted for 100 ms. Following a blank screen (400 ms), the second facial emotional change video (S2) appeared for 600 ms. Finally, a blank screen terminated the trial, which lasted for 1000–2000 ms. The participants were asked to judge the identity of the face in the videos during the 1000–2000 ms blank interval after S2. The S1 and S2 videos were the same expression change in each trial. Participants completed two blocks of 90 trials, yielding a total of 180 trials per participant (60 angry-neutral facial emotional change trials, 60 neutral-angry facial emotional change trials, and 60 neutral-neutral facial emotional change trials). There were equal numbers of same and different trials. Before the experiment, participants received instructions and performed a block of six practice trials that included two practice trials for each expression change condition.

### EEG Recording and Analysis

Electroencephalogram (EEG) was recorded from 64 scalp sites using the BrainAmps system (Brain Products, Munchen, Germany). The vertical electrooculogram (EOG) was recorded with electrodes placed below the right eye and the horizontal EOG was recorded from the right orbital rim. Electrode impedance was held below 5 kΩ. The EEG and EOG activities were amplified using a DC-100 Hz bandpass and were continuously sampled at 500 Hz/channel.

For preprocessing the ERP analysis, the EEG data were recomputed to average mastoid reference. The data were further filtered off-line (0.05–30 Hz bandwidth). Ocular artifacts (blinks and other movements) were corrected using a Gratton and Coles-based algorithm off-line. Trials with artifacts exceeding 100 μV mainly due to amplifier clippings and peak-to-peak deflections were omitted from the averaging. The EEG data were segmented from −200 ms to 1000 ms relative to the second facial emotional change video (S2), with a 200 ms pre-stimulus baseline. Based on previous studies (Luo et al., 2010; Caharel et al., 2011; Ran et al., 2014c), the P100 component (80–130 ms) was analyzed at O1/O2, P3/P4, and PO3/PO4 electrodes. In addition, the N170 component was analyzed within a time frame of 130–190 ms post stimulus onset at P5/P6, P7/P8, and PO7/PO8 electrodes. The P200 component (190–250 ms) was determined over O1/O2, P3/P4, PO3/PO4, and PO7/PO8 electrodes. Peak amplitudes and latencies of these components were subjected to repeated-measures ANOVA with facial emotional changes (angry-neutral, neutral-angry, vs. neutral-neutral) and hemisphere (right vs. left) as within-participant factors and with social anxiety (high vs. low) as a between-participant factor.

Given the absence of a sharply defined peak (Zhang et al., 2006; Van Beek et al., 2014), the LPP (300–700 ms) component was observed and quantified as mean amplitudes at the following sites: C3/C4, P3/P4, and Cz/Pz electrodes (Hagemann et al., 2016). Mean amplitudes of the LPP component were entered into a 3 × 3 × 2 ANOVA with the within-participant factors facial emotional changes, hemisphere (left, middle, vs. right), and the between-participant factor social anxiety. When appropriate, degrees of freedom for the repeated-measures factors were corrected according to Greenhouse–Geisser.

## Results

### Behavioral Results

A repeated-measure ANOVA with facial emotional changes (angry-neutral, neutral-angry, vs. neutral-neutral) as a within-participant factor, and social anxiety (high vs. low) as a between-participant factor was calculated based on participants’ accuracy and reaction time (RT) data. The mean accuracy and RT for each condition are displayed in **Table 1**.

**Table 1:** Means and standard deviations of accuracy and reaction time (RT) for HSA (high socially anxious) and LSA (low socially anxious) group in each facial emotional change condition.

Social Anxiety	Facial Emotional Changes	Accuracy (%)		Response time (ms)	
		*M*	*SD*	*M*	*SD*
LSA	neutral–neutral	94.29	5.42	417.43	98.25
	angry–neutral	84.64	7.66	419.69	126.09
	neutral–angry	84.05	9.71	459.26	150.75
HSA	neutral–neutral	92.05	5.41	403.33	129.83
	angry–neutral	87.44	7.92	414.80	182.01
	neutral–angry	83.08	11.60	440.86	182.98

The analysis of the accuracy data showed a main effect for facial emotional changes [*F*(2,50) = 23.50, *p* < 0.001, $\eta_{p}^{2}$ = 0.484]. *Post hoc t*-tests showed that the accuracy rates in the angry-neutral facial emotional change condition were lower (*M* = 86.04%, *SD* = 7.77%) than those in the neutral-neutral (*M* = 93.17%, *SD* = 5.43%) facial emotional change condition [*t*(26) = −5.31, *p* < 0.001]. However, the differences in accuracy rates between the angry-neutral (*M* = 86.04%, *SD* = 7.77%) and neutral-angry (*M* = 83.57%, *SD* = 10.47%) facial emotional change trials failed to reach significance [*t*(26) = 1.68, *p* = 0.104]. With regard to the RT data, no significant effects were found (all *Fs* < 3.17, *ps* > 0.051).

To see if S1 had an influence on S2, we compared the accuracy and RT data in same vs. different trials. We found that the accuracy rates in the same trial (*M* = 96.91%, *SD* = 2.99%) were higher than those seen in the different (*M* = 76.12%, *SD* = 13.70%) trial [*t*(26) = 7.57, *p* < 0.001). The RTs in the same trial were shorter (*M* = 398.30 ms, *SD* = 132.16 ms) than those seen in the different (*M* = 471.30 ms, *SD* = 176.45 ms) trial [*t*(26) = −2.95, *p* = 0.007]. We also compared the accuracy and RT data in women vs. men facial trials to see if sex of the face stimuli had an impact. No effect reached significance [accuracy: *t*(26) = 1.02, *p* = 0.319; RTs: *t*(26) = 0.40, *p* = 0.691].

### Electrophysiological Results

#### P100

The ANOVA of P100 amplitude showed that no main effects or interactions reached significance [*facial emotional changes*: *F*(2,34) = 0.49, *p* = 0.616, $\eta_{p}^{2}$ = 0.019; *hemisphere*: *F*(1,25) = 1.93, *p* = 0.177, $\eta_{p}^{2}$ = 0.072; *social anxiety*: *F*(1,25) = 0.63, *p* = 0.434, $\eta_{p}^{2}$ = 0.025; *hemisphere* × *facial emotional changes*: *F*(2,43) = 0.33, *p* = 0.688, $\eta_{p}^{2}$ = 0.013; *hemisphere* × *social anxiety*: *F*(1,25) = 1.99, *p* = 0.171, $\eta_{p}^{2}$ = 0.074; *facial emotional changes* × *social anxiety*: *F*(2,34) = 0.75, *p* = 0.433, $\eta_{p}^{2}$ = 0.029; *facial emotional changes* × *hemisphere* × *social anxiety*: *F*(2,43) = 0.09, *p* = 0.887, $\eta_{p}^{2}$ = 0.004].

**FIGURE 3:**
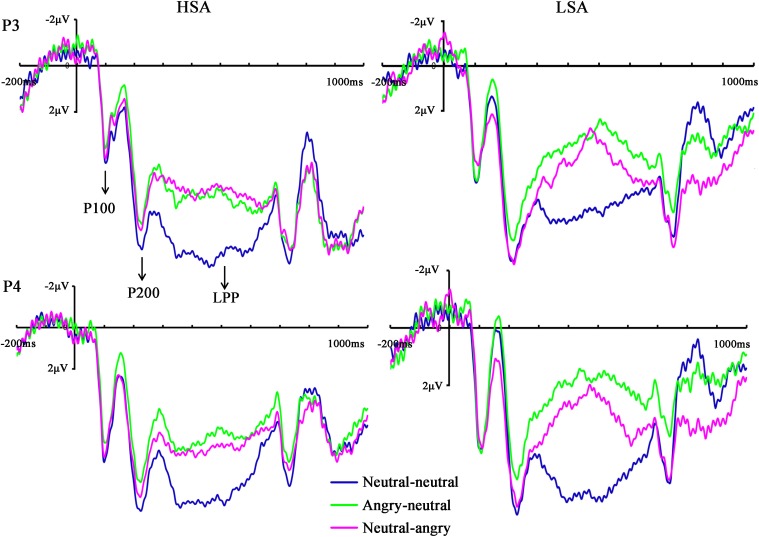
Grand mean ERPs in each facial emotional change condition at the left (P3) and right (P4) hemisphere electrodes with time windows of P100, P200, and LPP for HSA and LSA participants.

The same ANOVA on P100 latency revealed that none of the main effects or interactions were significant [*facial emotional changes*: *F*(2,35) = 0.06, *p* = 0.881, $\eta_{p}^{2}$ = 0.002; *hemisphere*: *F*(1,25) < 0.001, *p* = 0.991, $\eta_{p}^{2}$ < 0.001; *social anxiety*: *F*(1,25) = 0.48, *p* = 0.496, $\eta_{p}^{2}$ = 0.019; *hemisphere* × *facial emotional changes*: *F*(2,45) = 1.11, *p* = 0.332, $\eta_{p}^{2}$ = 0.043; *hemisphere* × *social anxiety*: *F*(1,25) = 0.39, *p* = 0.537, $\eta_{p}^{2}$ = 0.015; *facial emotional changes* × *social anxiety*: *F*(2,35) = 2.82, *p* = 0.090, $\eta_{p}^{2}$ = 0.101; *facial emotional changes* × *hemisphere* × *social anxiety*: *F*(2,45) = 1.79, *p* = 0.182, $\eta_{p}^{2}$ = 0.067] (**Figure 3**).

#### N170

**FIGURE 4:**
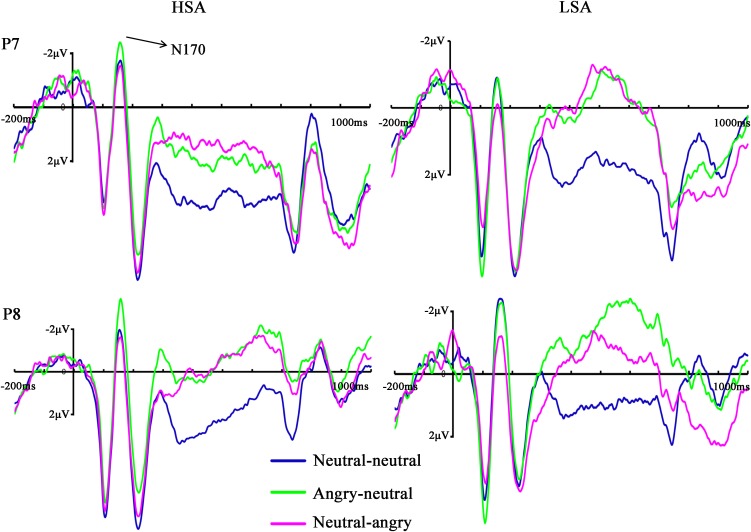
Grand mean ERPs in each facial emotional change condition at the left (P7) and right (P8) hemisphere electrodes with time window of N170 for HSA and LSA participants.

**FIGURE 5:**
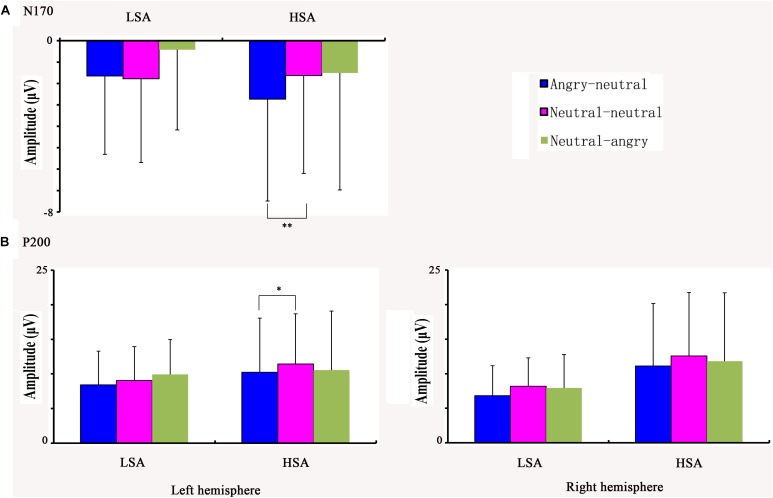
The average N170 peaks in each facial emotional change condition for HSA and LSA participants **(A)**. The average P200 peaks in each facial emotional change condition for HSA and LSA participants at the left and right hemisphere electrodes **(B)** (^∗^*p* < 0.05, ^∗∗^*p* < 0.01).

The ANOVA of N170 amplitude revealed a significant main effect of facial emotional changes [*F*(2,45) = 10.96, *p* < 0.001, $\eta_{p}^{2}$ = 0.305]. *Post hoc t*-tests showed that the amplitudes (less negative amplitudes) found in the neutral-angry (*M* = −0.95 μV, SD = 4.59 μV) facial emotional change trial were smaller than those seen in the angry-neutral (*M* = −2.17 μV, *SD* = 4.17 μV) facial emotional change trial [*t*(26) = 5.64, *p* < 0.001], while no differences between the neutral-neutral (*M* = −1.70 μV, *SD* = 4.16 μV) and angry-neutral (*M* = −2.17 μV, *SD* = 4.17 μV) facial emotional change trials were observed [*t*(26) = 1.70, *p* = 0.101]. Furthermore, there was a significant interaction between social anxiety and facial emotional changes [*F*(2,45) = 3.63, *p* = 0.039, $\eta_{p}^{2}$ = 0.127]. Follow-up analyses confirmed the that amplitudes (more negative amplitudes) in the angry-neutral facial emotional change trial were larger than those seen in the neutral-neutral facial emotional change trial for HSA participants [angry-neutral: *M* = –2.73 μV, *SD* = 4.76 μV; neutral-neutral: *M* = –1.63 μV, *SD* = 4.57 μV; *t*(12) = –3.28, *p* = .007] but not for LSA [angry-neutral: *M* = –1.65 μV, *SD* = 3.66 μV; neutral-neutral: *M* = –1.78 μV, *SD* = 3.91 μV; *t*(13) = 0.32, *p* = 0.751] participants (**Figures 4**, **5A**).

The same ANOVA was performed on N170 latency. We observed a significant main effect of facial emotional changes [*F*(2,43) = 4.60, *p* = 0.019, $\eta_{p}^{2}$ = 0.156]. *Post hoc t*-tests showed that the peak latency was significantly longer in the angry-neutral facial emotional change trial (*M* = 156.79 ms, *SD* = 13.21 ms) than those seen in the neutral-neutral (*M* = 153.64 ms, *SD* = 12.82 ms) case [*t*(26) = −3.63, *p* = 0.001]. However, the differences of peak latency between the angry-neutral (*M* = 156.79 ms, *SD* = 13.21 ms) and neutral-angry (*M* = 154.37 ms, *SD* = 13.10 ms) facial emotional change trials failed to reach significance [*t*(26) = −1.95, *p* = 0.062]. No other effect reached significance (all *F*s < 2.09, *p*s > 0.161).

#### P200

The ANOVA of posterior P200 amplitude showed a main effect of facial emotional changes [*F*(2,43) = 7.20, *p* = 0.003, $\eta_{p}^{2}$ = 0.224]. *Post hoc t*-tests revealed that the P200 amplitudes were smaller in the angry-neutral facial emotional change condition (*M* = 9.10 μV, *SD* = 6.66 μV) than those seen in the neutral-neutral angry-neutral facial emotional change condition (*M* = 10.25 μV, *SD* = 6.54 μV) case [*t*(26) = 4.21, *p* < 0.001], while no differences between the neutral-neutral (*M* = 10.25 μV, *SD* = 6.54 μV) and neutral-angry (*M* = 10.02 μV, *SD* = 7.31 μV) facial emotional change trials were found [*t*(26) = −0.61, *p* = 0.549]. In addition, there was a significant interaction between social anxiety and hemisphere [*F*(1,25) = 6.20, *p* = 0.02, $\eta_{p}^{2}$ = 0.199]. The increased P200 amplitudes were observed over the left hemisphere (*M* = 9.13 μV, *SD* = 4.84 μV) when compared with the right (*M* = 7.66 μV, *SD* = 4.26 μV) hemisphere for LSA participants [*t*(13) = 3.00, *p* = 0.010]. More importantly, a significant three-way interaction among facial emotional changes, social anxiety, and hemisphere was observed [*F*(2,47) = 3.38, *p* = 0.045, $\eta_{p}^{2}$ = 0.119]. Further analyses suggested that P200 amplitudes at the left hemisphere were reduced in the angry-neutral facial emotional change trial when compared with the neutral-neutral facial emotional change trial for HSA participants [angry-neutral: *M* = 10.23 μV, SD = 7.83 μV; neutral-neutral: *M* = 11.44 μV, *SD* = 7.24 μV; *t*(12) = −2.35, *p* = 0.037] but not for LSA participants [(angry-neutral: *M* = 8.43 μV, *SD* = 4.85 μV; neutral-neutral: *M* = 9.05 μV, *SD* = 4.88 μV; *t*(13) = −1.83, *p* = 0.090] (**Figure 5B**).

The same ANOVA when performed on posterior P200 latency showed no significant main effects or interactions [*facial emotional changes*: *F*(2,46) = 0.02, *p* = 0.972, $\eta_{p}^{2}$ = 0.001; *hemisphere*: *F*(1,25) = 0.08, *p* = 0.785, $\eta_{p}^{2}$ = 0.003; *social anxiety*: *F*(1,25) = 0.28, *p* = 0.604, $\eta_{p}^{2}$ = 0.011; *hemisphere* × *facial emotional changes*: *F*(2,49) = 0.48, *p* = 0.619, $\eta_{p}^{2}$ = 0.019; *hemisphere* × *social anxiety*: *F*(1,25) = 0.48, *p* = 0.494, $\eta_{p}^{2}$ = 0.019; *facial emotional changes* × *social anxiety*: *F*(2,46) = 0.01, *p* = 0.996, $\eta_{p}^{2}$ < 0.001; *facial emotional changes* × *hemisphere* × *social anxiety*: *F*(2,49) = 1.57, *p* = 0.217, $\eta_{p}^{2}$ = 0.059].

#### LPP

**FIGURE 6:**
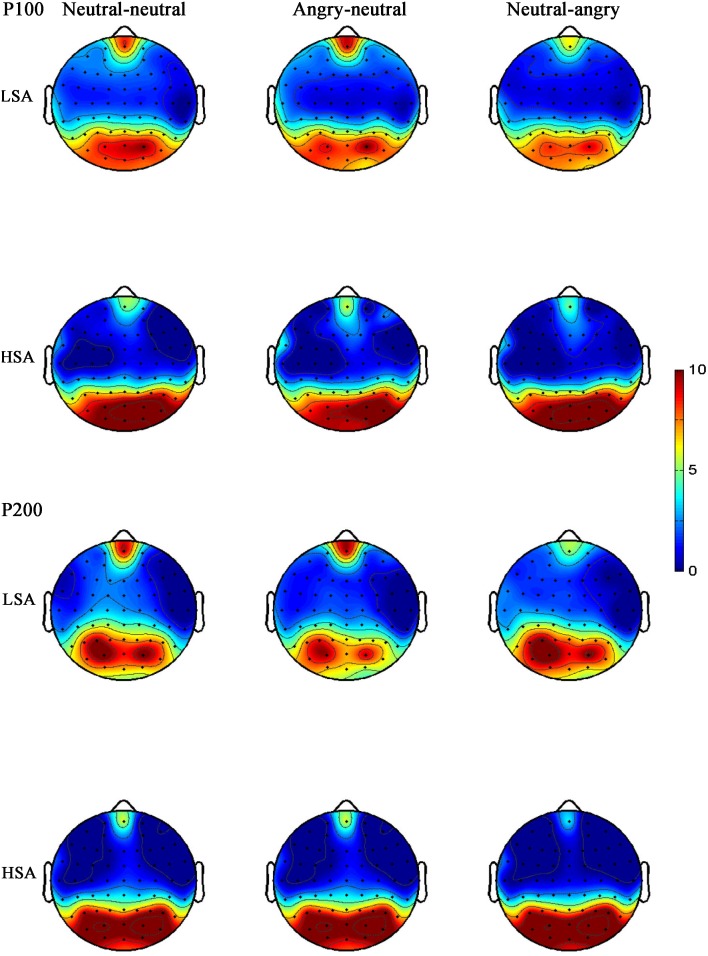
Grand-averaged ERP topographies of the P100 and P200 components for HSA and LSA participants in each facial emotional change condition.

**FIGURE 7:**
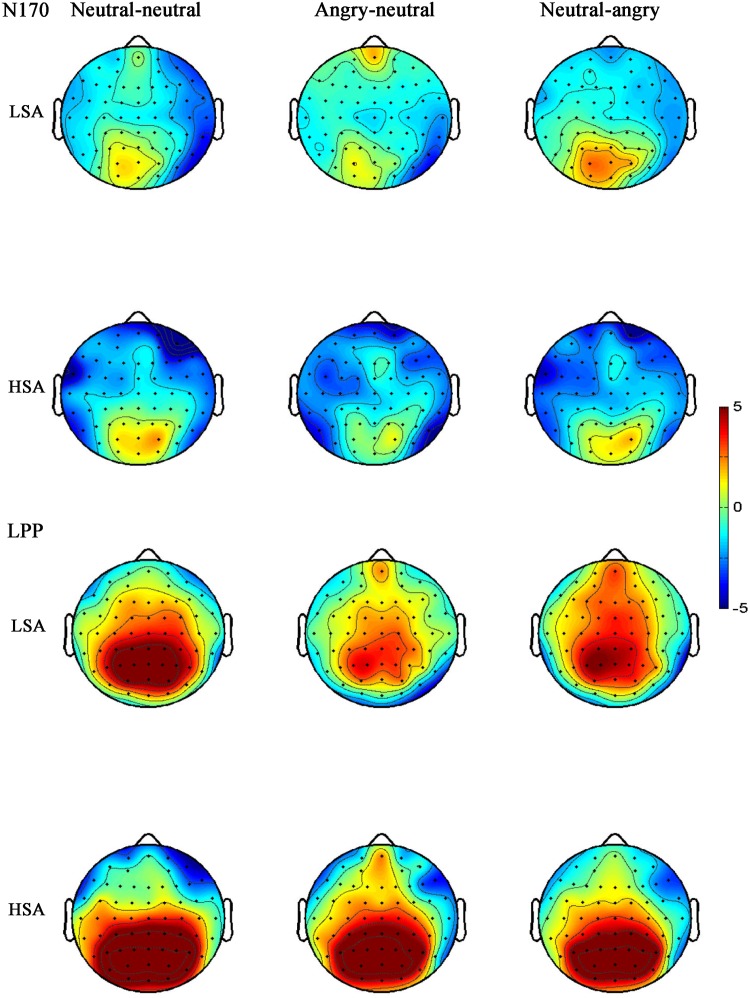
Grand-averaged ERP topographies of the N170 and LPP components for HSA and LSA participants in each facial emotional change condition.

The ANOVA for LPP mean amplitudes yielded a significant main effect of facial emotional changes [*F*(2,41) = 4.33, *p* = 0.019, $\eta_{p}^{2}$ = 0.147]. *Post hoc t*-tests revealed that the amplitudes were smaller in the neutral-angry [*M* = 3.21 μV, *SD* = 2.95 μV; *t*(26) = 2.71, *p* = 0.012] and angry-neutral facial emotional change trials [*M* = 3.72 μV, *SD* = 2.69 μV; *t*(26) = 2.87, *p* = 0.008] than those seen in the neutral-neutral [*M* = 4.90 μV, *SD* = 2.44 μV] case. No other significant amplitude differences were found for the LPP component (all *F*s < 3.77, *ps* > 0.064). The grand average ERP waveforms and topographies of the P100, N170, P200, and LPP components in each condition are shown in **Figures 3**, **4**, **6**, **7**.

## Discussion

The present study employed the S1-S2 paradigm that some researchers had adopted previously (Ran et al., 2014a, c; Li et al., 2017) to explore the perception of facial emotional changes in individuals with social anxiety. Our behavioral results demonstrated lower accuracy rates in the angry-neutral facial emotional change trial than those seen in the neutral-neutral case. For the ERP results, we found that the N170 amplitudes were significantly larger for angry-neutral facial emotional changes than those for the neutral-neutral case only for HSA participants. However, it was found that the P200 left hemisphere amplitudes were reduced in the angry-neutral facial emotional change trial when compared with the neutral-neutral facial emotional change trial for HSA participants but not for LSA participants. Finally, the LPP amplitudes in the neutral-angry and angry-neutral facial emotional change trials were smaller than those in the neutral-neutral facial emotional change trials.

The present study revealed lower accuracy rates for angry-neutral facial emotional changes than those for the neutral-neutral facial emotional changes, suggesting an inferior processing of angry-neutral facial emotional changes. Considering the fact that the angry-neutral facial emotional changes are morphed (ambiguous) negative expressions (Gutiérrez-García and Calvo, 2017), people may be more cautious towards them. However, there are other studies to show that people have better performances (better memory) for emotional faces when compared with neutral faces, which is inconsistent with our finding (Grady et al., 2007). While caution should be taken in interpreting these results, one reason for the discrepancy might be that different participants were recruited in our study (HSA and LSA participants elevated on the social anxiety scale) and in the study conducted by Grady et al. (2007) (adults without a history of anxiety disorders).

The N170 component is thought to reflect the analytical processing of different facial elements (eyes, mouth, etc.) (Peschard et al., 2013). The present study found that HSA participants showed larger N170 amplitudes in angry-neutral facial emotional change trials than those in the neutral-neutral case. Such enhanced N170 amplitudes indicate that HSA participants may engage in more analytical processing of different facial elements when viewing the angry-neutral facial emotional changes. As HSA participants usually experience social inhibition, they have less experience with social threat stimuli (Ran and Chen, 2017). There has been a growing recognition that less experience with social stimuli leads to a relatively more feature-based processing of these stimuli (Hugenberg et al., 2007; Short and Mondloch, 2013). Similar to our finding, larger N170 amplitudes were observed when persons clinically diagnosed with social phobia (social phobic persons) processed static angry faces in an emotion identification task (Kolassa and Miltner, 2006).

Interestingly, the P200 was functionally associated with the evaluation of emotional relevance of a visual stimulus, as reported by many studies previously (Kolassa et al., 2009; Peschard et al., 2013). The P200 amplitudes were decreased in the angry-neutral facial emotional change trials when compared with neutral-neutral facial emotional change trials for HSA participants but not for LSA participants, which suggests that HSA participants may have difficulties in processing emotions when they encounter angry-neutral facial emotional changes. This is well in line with evidence from studies on emotional processes that indicate the deficient emotion evaluation in high anxious subjects (e.g., Rossignol et al., 2005). The difficulties with emotion evaluation in HSA participants might arise from their distorted appraisals of social situations, which is corroborated by the fact that these participants tend to show inaccurate interpretations of the self (socially incompetent) and others (critical judges) (Goldin et al., 2009). However, increased P2 amplitudes were reported in HSA participants for static angry faces (Ran and Chen, 2017), which was inconsistent with our findings. Such inconsistency suggests that facial expression (dynamic and static expressions) may moderate N170 in HSA participants. This needs to be examined in further studies.

The present result found smaller LPP amplitudes in the neutral-angry facial emotional change trial than those in the neutral-neutral case. This is similar to the previous ERP studies (Yuan et al., 2007; Chen and Luo, 2010), which demonstrated that reduced LPP (or P3) amplitudes were elicited by static negative stimuli. However, some other studies found that negative pictures evoked enhanced LPP amplitudes (Moser et al., 2008; Wieser and Moscovitch, 2015). Yuan et al. (2007) suggested that the reduced LPP (or P3) amplitudes under negative condition were due to the fact that participants needed to overcome the emotional negativity bias that favors the processing of negatively valenced information in implicit emotional experiments. In addition, the LPP amplitudes in the angry-neutral facial emotional change trials were smaller than those in the neutral-neutral facial emotional change trials. This suggests that the differences between the neutral-angry and angry-neutral facial emotional changes were not observed at a later stage of processing. However, the N170 (an early component) amplitudes were found to be smaller in the neutral-angry facial emotional change trials than those in the angry-neutral case.

Some studies have employed the S1-S2 paradigm to explore the recognition of emotional faces (Ran et al., 2014a,c; Li et al., 2017). Given that the processing of emotion was implicit (judging the identity of the faces or identifying the gender of the faces) in these studies, such task manipulation is not optimal. It would be nice to adopt an explicit task of emotion (judging the expression/expression change) in further research.

## Conclusion

In the present study, the ERP measure was used to explore the perception of facial emotional changes in individuals with social anxiety. The behavioral data showed lower accuracy rates in the angry-neutral facial emotional change trial than those in the neutral-neutral case, indicating an inferior processing of the angry-neutral facial emotional change. The electrophysiological data showed larger N170 amplitudes in the angry-neutral facial emotional change trial than those in the neutral-neutral case for HSA participants, implying that they might be engaged in more analytical processing of different facial elements. However, HSA participants showed smaller P200 amplitudes in the angry-neutral facial emotional change trial when compared with the neutral-neutral case, which suggested that they might have difficulties in processing emotions when they encounter angry-neutral facial emotional changes. Finally, we found that the LPP amplitudes in the neutral-angry and angry-neutral facial emotional change trials were smaller than those in the neutral-neutral facial emotional change trials, regardless of the social anxiety. In short, we suggest that the effect of social anxiety on the facial emotional changes may change over time.

## Author Contributions

QZ designed the experiments. QZ and GR collected and analyzed the data. QZ primarily wrote the manuscript. All authors discussed the results and commented on the manuscript.

## Conflict of Interest Statement

The authors declare that the research was conducted in the absence of any commercial or financial relationships that could be construed as a potential conflict of interest.
